# Fault-Free Protection Level Equation for CLAS PPP-RTK and Experimental Evaluations

**DOI:** 10.3390/s22093570

**Published:** 2022-05-07

**Authors:** Euiho Kim, Jaeyoung Song, Yujin Shin, Saekyul Kim, Pyo-Woong Son, Sulgee Park, Sanghyun Park

**Affiliations:** 1 Department of Mechanical & System Design Engineering, Hongik University, Seoul 04006, Korea; 2 Maritime PNT Research Office, KRISO, Daejeon 34103, Korea; justin2028@kriso.re.kr (J.S.); sgpark@kriso.re.kr (S.P.); shpark@kriso.re.kr (S.P.); 3 Research Institute of Science and Technology, Hongik University, Seoul 04006, Korea; syj3558@naver.com; 4 Department of Mechanical Engineering, Hongik University, Seoul 04006, Korea; idea2693@naver.com; 5 Korea Research Institute of Ships and Ocean Engineering, Daejeon 34103, Korea; pwson@kriso.re.kr; 6 Department of Ship and Ocean Engineering, University of Science and Technology (UST), Daejeon 34113, Korea

**Keywords:** GNSS precise positioning, protection levels, integrity, PPP-RTK

## Abstract

Centimeter level augmentation system (CLAS) of the quasi-zenith satellite system (QZSS) is the first precise point positioning-real time kinematic (PPP-RTK) augmentation system of the global navigation satellite system (GNSS), which is currently providing services for Japan. CLAS broadcasts the state-space representation of correction messages along with integrity messages regarding satellite faults and the quality index of each correction. In other GNSS augmentation systems, such as the space-based augmentation system (SBAS) of GNSS, the quality indices of correction messages are used to generate fault-free protection levels that represent a position bound containing a true user position with a probability of missed detections. Although the protection level equations are well defined for the SBAS, a protection level equation for the CLAS PPP-RTK service has not been rigorously discussed in the literature. This paper proposes a fault-free protection level equation for the PPP-RTK methods that considers the probability of correct integer ambiguity fixes in the GNSS carrier phase measurements as well as the CLAS correction quality messages. The computed protection levels with position errors were experimentally compared by processing the GNSS measurements from the GNSS Earth Observation Network (GEONET) stations in Japan and the L6 messages from the CLAS broadcast using the virtual reference station-real time kinematic (VRS-RTK) techniques. Our results, based on the GEONET dataset spanning 7 days, showed that the computed protection levels using the proposed equations were larger than the position errors for all epochs. In the dataset, the RMS errors of the CLAS VRS-RTK position were 4.6 and 14 cm in the horizontal and vertical directions, respectively, whereas the horizontal protection levels ranged from 25 cm to 2.3 m and the vertical protection levels ranged from 50 cm to 5.2 m based on fault-free integrity risk of $10^{-7}$.

## 1. Introduction

The performance of the global navigation satellite system (GNSS) is enhanced by differential GNSS techniques, including ground- and space-based augmentation systems (GBAS and SBAS) of the global positioning system (GPS) [1,2]. The GBAS and SBAS were primarily developed to guide aircraft navigation, and their design approach and operational philosophies centered on system safety. The safety levels of GBAS and SBAS are quantized as system integrity measures, and one of the important integrity measures is a protection level [3,4]. The protection level represents the bound of the true position error at the risk of a missed-detection probability. In GBAS and SBAS, a position bound is computed using the well-defined protection level equations that transform the range-domain Gaussian error distributions of each visible satellite to the position-domain Gaussian error distributions via a user-to-satellite geometry. The range-domain error distributions used in the protection level equations are determined by the GBAS and SBAS service providers and are broadcast to users as quality indicators associated with each correction message. An important characteristic of the range domain error distribution is to overbound the actual error distribution of the correction messages in order for the protection levels, computed using the quality indicators, to overbound the actual user position errors with a missed-detection probability. Consequently, studies have been conducted on various overbounding techniques based on probability density function (PDF), cumulative distribution function (CDF), and paired overbounding [5,6,7,8].

Centimeter level augmentation system (CLAS) of the quasi-zenith satellite system (QZSS) is a very recent GNSS augmentation system of Japan [9,10,11]. Unlike the GBAS and SBAS, which primarily use the GNSS code phase measurements as ranging sources, CLAS is a precise point positioning–real-time kinematic (PPP-RTK) augmentation system that allows users to resolve integer ambiguities in carrier phase measurements within several minutes by using the CLAS correction messages. Therefore, the CLAS users can use the carrier phase as a ranging source and achieve precise positioning similar to that of RTK. CLAS also provides quality indicators (also called integrity messages) for each correction message so that the users can compute the protection levels. However, the CLAS protocol currently does not specify any forms of protection-level equations, and quality message generation schemes are not well known [12,13]. Owing to lack of information, the CLAS users are not encouraged to rely on the quality messages for safe navigation.

Unfortunately, the protection level equations of GBAS or SBAS cannot be directly applied to the CLAS PPP-RTK position solutions, because GBAS or SBAS do not use the carrier phase as ranging sources. Previous studies have proposed protection-level equations for PPP and RTK that use the carrier phase as ranging sources [14,15,16,17,18,19]. These studies were based on receiver autonomous integrity monitoring (RAIM) [20], and their protection levels were derived to protect against the hard-to-detect faults in ranging measurements and cycle slips. Feng proposed a Kalman filter-based RAIM for the carrier phase [14]. In this approach, the de-correlated innovations of the Kalman filter were used to detect faults. Protection levels were computed either from the covariance matrix of the Kalman filter or from using the geometry of the satellite whose faults would be most difficult to detect. References [15,16] proposed isotropy-based protection levels (IBPL), particularly designed for the PPP position solutions. Assumptions regarding the behavior of ranging errors in terms of their size, direction, and protection levels as derived from a multivariate t-distribution of measurement errors were not made in IBPL. Ahmed et al. proposed protection levels for RTK, which were modelled in a modified form from the solution-separation RAIM methods [17,18]. An integrity risk in this approach considers the mutually exclusive events of correct and incorrect ambiguity resolutions in the least-squares ambiguity decorrelation adjustment (LAMBDA) method, which was introduced in [19]. Although these studies can be applied to derive protection levels for the CLAS PPP-RTK position solutions, their protection levels would be relatively larger because they do not use the CLAS integrity messages or the fault monitoring capability of the CLAS network.

This paper proposes a protection-level equation for the PPP-RTK services broadcasting quality messages. Because both PPP-RTK and SBAS broadcast state space representation (SSR) of the correction messages, our proposed protection level equations are based on the SBAS protection level equations and are extended to reflect virtual reference station (VRS)-RTK positioning scheme, which is a method of processing the PPP-RTK correction messages on the user side. Additionally, the integrity messages of the CLAS service are assumed to have a standard deviation of a Gaussian distribution, similar to the SBAS. This study compared the protection levels computed using the CLAS integrity messages with the VRS-RTK position errors. For the VRS-RTK process, we used CLAS L6 messages and GNSS observation measurements from the GNSS Earth Observation Network (GEONET) station in Japan. The L6 integrity messages were only used to compute the protection levels, because they were significantly inflated from the actual error distribution of the correction messages; however, the VRS-RTK process used the correction error distributions used in practice, whose standard deviation was significantly smaller than that of the integrity messages.

This paper provides an overview of the architecture of CLAS and broadcasting messages in Section 2. Section 3 discusses the proposed protection-level equations for PPP-RTK. Section 4 presents examples of broadcast integrity indices and computed horizontal and vertical protection levels with the dataset. Finally, the discussion and conclusions are presented.

## 2. Overview of the CLAS Broadcast Messages

CLAS receives the GNSS observation data from approximately 1200 GEONET stations and processes the data to generate the correction and integrity messages in a Compact SSR format. The Compact SSR messages are broadcast through the L6 band by the QZSS and are defined as RTCM 3 compatible proprietary message type 4073 [13]. The Compact SSR messages have 12 subtypes consisting of correction and integrity information, and the message types are listed in Table 1 [21]. The correction messages for the orbit, clock, code bias, and phase bias are called common corrections. The zenith tropospheric delay and slant ionospheric delay for each GNSS are referred to as local corrections.

Among the message subtype IDs, the quality messages are included in subtype IDs 7, 8, 9, and 12. Subtype ID 7 provides a quality indicator for the user range accuracy (URA) of each satellite, and others provide a troposphere quality indicator as well as an SSR slant total electron content (STEC) quality indicator. The quality indicator is represented by a combination of CLASS and VALUE, the values of which range from 0 to 7, as seen in Equation (1). The SSR URA and tropospheric quality indicators were converted to a physical quantity using the following Equation [21]:

(1) $$\mathrm{Quality}\;\mathrm{Indicator}\;\left[\mathrm{mm}\right]\leq 3^{\mathrm{CLASS}\;}\left(1+\frac{\;\mathrm{VALUE}\;}{4}\right)-1\;\left[\mathrm{mm}\right]$$

The SSR STEC physical quantity was read from a table relating the SSR STEC quality indicators to the SSR STEC correction uncertainty.

The total ranging error for *i*th satellite can be estimated by the following equation

(2) $$\sigma_{i}=\sqrt{{\left(\sigma_{i,user}\right)}^{2}+{\left(\sigma_{i,sis}/10\right)}^{2}+{\left(\frac{40.3\times 10^{16}}{f^{2}}\sigma_{i,\;\mathrm{iono}\;}\times 100\right)}^{2}+{\left(\left(\sigma_{i,\;\mathrm{trop}\;}/10\right)/\sin E_{i}\right)}^{2}},$$

where $\sigma_{i,user}$ is a user-specific local error, such as multipath errors. $\sigma_{i,sis}$ is a signal in space error representing the correction message uncertainty of the orbit, clock, code, and carrier biases. $\sigma_{i,\;\mathrm{iono}\;}$ is the ionospheric delay correction uncertainty provided from the STEC correction quality indicator in total electron content unit (TECU). $\sigma_{i,\;\mathrm{trop}\;}$ represent the troposphere delay correction uncertainty. f is the frequency of the GNSS signal and $E_{i}$ is the satellite elevation angle. The total ranging error is calculated in centimeters, which is why both $\sigma_{i,sis}$ and $\sigma_{i,\;\mathrm{trop}\;}$divided by 10 is used, and the same applies for $\sigma_{i,\;\mathrm{iono}}$. Some examples of the CLAS broadcast quality indicators are presented in Section 4.

## 3. Proposed Fault-Free Protection Level Equations for CLAS PPP-RTK Service

To develop a fault-free protection-level equation for a PPP-RTK system, the current protection-level equations of SBAS are used as the baseline equations because both SBAS and PPP-RTK broadcast the correction and quality messages in SSR formats. This section provides an overview of the SBAS fault-free protection level equation, followed by the proposed PPP-RTK protection level equation.

### 3.1. Fault-Free Protection Level Equations of SBAS

The SBAS L1 frequency-only protection-level equation uses the broadcast correction message uncertainties and user-defined multipath and noise uncertainties. For an individual SBAS pseudo range error, the combined range error variance for the *i*th satellite was constructed as follows:

(3) $$\sigma_{i}^{2}=\sigma_{flt,i}^{2}+\sigma_{UIRE,i}^{2}+\sigma_{tropo,i}^{2}+\sigma_{air,i{}^{\prime }}^{2}$$

where $\sigma_{flt,i}^{2}$ is the variance of the fast and long-term satellite clock error corrections. $\sigma_{UIRE,i}^{2}$ is the variance of the user ionosphere range error correction, and $\sigma_{tropo,i}^{2}$ is the variance of the tropospheric error correction, and $\sigma_{air,i}^{2}$ is the variance of the multipath error excluding multipath from ground. It should be noted that each variance is determined from a zero-mean Gaussian distribution that overbounds a non-ideal Gaussian distribution of correction errors.

The pseudo range variance is used as a weighting matrix to compute an SBAS position solution such that

(4) $$W=\left[\begin{array}{cccc} \frac{1}{\sigma_{1}^{2}} & 0 & \ldots & 0\\ 0 & \frac{1}{\sigma_{2}^{2}} & \cdots & 0\\ \vdots & \vdots & \ddots & 0\\ 0 & 0 & \ldots & \frac{1}{\sigma_{n}^{2}} \end{array}\right].$$

The direction vector from a user to satellite can be formulated as

(5) $$G_{i}=\left[\cos\left(El_{i}\right)\sin\left(Az_{i}\right)\;\;\cos\left(El_{i}\right)\cos\left(Az_{i}\right)\;\;\;\sin\left(El_{i}\right)\;\;1\right].$$

Then, the standard deviation of the position estimate uncertainty in East/North/Up (ENU) coordinates is

(6) $$\sigma_{p}=\sqrt{{\left(G^{T}WG\right)}^{-1}}.$$

With a missed-detection probability and its corresponding Gaussian tail value of $K_{ffmd}$, the vertical protection level (VPL) bounding vertical position errors is

(7) $${\mathrm{VPL}}_{\mathrm{SBAS}}=K_{ffmd}\sqrt{{\left[{\left(G^{T}WG\right)}^{-1}\right]}_{\left(3,3\right)}}.$$

Similarly, the horizontal protection level (HPL) is computed.

### 3.2. Overview of Least-Squares Ambiguity Decorrelation Adjustment (LAMBDA)

The PPP-RTK service allows the use of the GNSS carrier phase for ranging measurements in a manner similar to a VRS-RTK process. A VRS-RTK process typically resolves the integer ambiguities in carrier phase measurements using the LAMBDA algorithm [22,23], and its upper bound probability of correctly fixing integer ambiguities is assessed using the integer bootstrapping method [24].

With GNSS measurements of the two receivers at a short baseline, the basic observation measurements are as follows:

(8) $$\Delta \nabla \rho =r+\varepsilon_{\rho },$$

(9) $$\Delta \nabla \Phi =r+\lambda_{f}N+\varepsilon_{\Phi },$$

where Δ∇ρ and Δ∇Φ are the double-difference code and carrier-phase measurements, respectively. r is the geometric range from a user to a satellite. $\lambda_{f}$ is the f frequency wavelength of a GNSS carrier. N is integer ambiguity. ε represents the uncorrected range measurements and noise. The linearized observation equation for a set of double-difference codes and carrier-phase measurements of visible satellites is

(10) y=Aa+Bb+ε,

where y is a vector of the double-difference code and carrier-phase measurements. a is a vector of integer ambiguities and A is a corresponding matrix with wavelengths as elements. B is a satellite geometry matrix, and b is the relative position vector from a VRS reference position to a GNSS receiver.

The popular LAMBDA method resolves a and computes the precise b in two steps. First, an unconstrained solution of Equation (10) is computed as

(11) $$\left[\begin{array}{c} \hat{b}\\ \hat{a} \end{array}\right]={\left(\left[\begin{array}{c} B^{T}\\ A^{T} \end{array}\right]Q_{y}^{-1}\left[\begin{array}{c} B\;\;A \end{array}\right]\right)}^{-1}\left[\begin{array}{c} B^{T}\\ A^{T} \end{array}\right]Q_{y}^{-1}y,$$

where $\hat{b}$ and $\hat{a}$ are the estimated baseline and float-integer ambiguities, respectively. $Q_{y}$ is the covariance matrix of ε in Equation (10). The covariance matrix of $\hat{b}$ and $\hat{a}$ is

(12) $$Q=\left[\begin{array}{cc} Q_{\hat{b}} & Q_{\hat{a}\hat{b}}\\ Q_{\hat{b}\hat{a}} & Q_{\hat{a}} \end{array}\right].$$

The second procedure of LAMBDA consists of the re-parameterization and search for a. The re-parameterization of the integer vector is implemented as follows:

(13) $$z=Za,\hat{z}=Z\hat{a},Q_{\hat{z}}=Z^{T}Q_{\hat{a}}Z,$$

where Z is the decorrelation transformation matrix. Then, the re-parameterized integer ambiguity is searched with respect to the following objective function:

(14) $$\underset{z}{min}{\left(\hat{z}-z\right)}^{T}Q_{\hat{z}}^{-1}\left(\hat{z}-z\right)$$

Once an optimal integer ambiguity vector, $\overset{ˇ}{z}$, is obtained from Equation (14), the original integer ambiguity vector estimate is obtained using the inverse of the transformation such that

(15) $$\overset{ˇ}{a}=Z^{-1}\overset{ˇ}{z}.$$

The presumed fixed baseline vector, $\overset{ˇ}{b}$ is

(16) $$\overset{ˇ}{b}=\hat{b}-Q_{\hat{b}\hat{a}}Q_{\hat{a}}^{-1}\left(\hat{a}-\overset{ˇ}{a}\right)$$

In addition, the covariance matrix of $\overset{ˇ}{b}$ is as follows:

(17) $$\begin{array}{c} Q_{\overset{ˇ}{b}}=Q_{\hat{b}}-Q_{\hat{b}\hat{a}}Q_{\hat{a}}^{-1}Q_{\hat{a}\hat{b}}\\ \;\;\;\;\;\;\;\;\;\;\;\;\;\;\;={\left(B^{T}\left(Q_{\Phi }^{-1}+Q_{\rho }^{-1}\right)B\right)}^{-1} \end{array},$$

where $Q_{\rho }$ and $Q_{\Phi }$ are the covariance matrices of the double-difference code and the carrier-phase measurements, respectively.

### 3.3. Proposed Fault-Free PPP-RTK Protection Level Equation

The integer ambiguity resolution through LAMBDA is a stochastic search process, and based on the bias-free estimates of float ambiguities, its upper bound probability of correct integer fixes is [24]

(18) $$\mathrm{Prob}\left(\overset{ˇ}{a}=a\right)=\prod_{i=1}^{n}\left(CDF\left(\frac{1}{2\sigma_{{\hat{a}}_{i|I}}}\right)-1\right)$$

where ${\hat{a}}_{i|I}$ is the conditional least-squares estimate of integer ambiguity. Because the correctness of the fixed integer ambiguities may significantly affect the position errors, the probability of Equation (18) must be considered in a protection-level equation. A protection level (XPL) and given fault-free risk probability ($I_{H0req}$) can be expressed as follows:

(19) $$\mathrm{Prob}\left\{\left|\hat{x}-x\right|>{\mathrm{XPL}}_{H0}\right\}=I_{H0req},$$

where $\hat{x}$ and x denote the estimated position and true position, respectively, in the horizontal or vertical directions. ${\mathrm{XPL}}_{H0}$ refers to the horizontal or vertical protection levels. Because the position error may exceed ${\mathrm{XPL}}_{H0}$ with correctly fixed (CF) or incorrectly fixed (IF) integer ambiguities, Equation (19) can be expanded to two conditional probabilities, as follows [9]:

(20) $$\begin{array}{c} \mathrm{Prob}\left\{\left|\hat{x}-x\right|>{\mathrm{XPL}}_{H0}\right\}=\mathrm{Prob}\left\{\left|\hat{x}-x\right|>{\mathrm{XPL}}_{H0}|\mathrm{CF}\right\}PCF\\ \;\;\;\;\;\;\;\;\;+\mathrm{Prob}\left\{\left|\hat{x}-x\right|>{\mathrm{XPL}}_{H0}|\mathrm{IF}\right\}PIF \end{array}$$

where PCF is the probability of the correct fix, which can be estimated from Equation (18). *PIF* is the probability of incorrect fix and PCF equals to 1−PIF.

Considering $\mathrm{Prob}\left\{\left|\hat{x}-x\right|>{\mathrm{XPL}}_{H0}|\mathrm{IF}\right\}=1$ as a conservative approach, the fault-free risk probability presuming that integer ambiguities are correctly fixed is

(21) $$\mathrm{Prob}\left\{\left|\hat{x}-x\right|>{\mathrm{XPL}}_{H0}|\mathrm{CF}\right\}=\frac{I_{H0req}-PIF}{1-PIF}.$$

Assuming that the position error follows a zero-mean Gaussian distribution, the corresponding tail value of Equation (21) was used as the $K_{ffmd}$ factor in Equation (7).

Using the total range error of CLAS, $\sigma_{i}$, in Equation (2), a weighting matrix for a single-differenced ranging source can be similarly constructed as in Equation (4). Because VRS-RTK uses double-difference code and carrier phase measurements, $Q_{\rho }$ and $Q_{\Phi }$ in Equation (17) can be expressed as

(22) $$Q_{\rho }=DW_{\rho }^{-1}D^{T}\;\mathrm{and}\;Q_{\Phi }=DW_{\Phi }^{-1}D^{T},$$

where D is the double-difference matrix. Using the $K_{ffmd}$ derived from Equation (21) and substituting Equation (23) into Equation (17), the fault-free horizontal and vertical protection level equations were computed as follows:

(23) $$\begin{array}{cc} \mathrm{HPL} & =K_{ffmd,\mathrm{HPL}}\sigma_{H}\;\;\;\;\;\;\;\;\;\;\;\;\;\;\;\;\\ & =K_{ffmd,\mathrm{HPL}}\sqrt{\frac{Q_{\overset{ˇ}{b}\left(1,1\right)}+Q_{\overset{ˇ}{b}\left(2,2\right)}}{2}+\sqrt{{\left(\frac{Q_{\overset{ˇ}{b}\left(1,1\right)}-Q_{\overset{ˇ}{b}\left(2,2\right)}}{2}\right)}^{2}+Q_{\overset{ˇ}{b}\left(1,2\right)}^{2}}}\;\mathrm{VPL}=K_{ffmd,\mathrm{VPL}}\sigma_{V}=K_{ffmd,\mathrm{VPL}}\sqrt{Q_{\overset{ˇ}{b}\left(3,3\right)}}. \end{array}$$

In Equation (23), $Q_{\overset{ˇ}{b}}$ is evaluated in ENU coordinates.

## 4. Evaluation of the Proposed Protection Levels for PPP-RTK

This section discusses the GNSS observation data and parsing process of the CLAS L6 broadcast messages used in this study. Subsequently, the resultant positioning error of the VRS-RTK positioning and computed protection levels are presented.

### 4.1. Experimental Data Processing

To evaluate the performance of the proposed protection level, we used the GNSS observation data provided by the website of the Geospatial Information Authority of Japan [25] and the L6 data provided by the website of the QZSS [26]. CLASLIB, an open-source software tool [26], was used to extract quality messages from the L6 broadcast data and generate the GNSS measurements for the VRS-RTK process.

**Figure 1:**
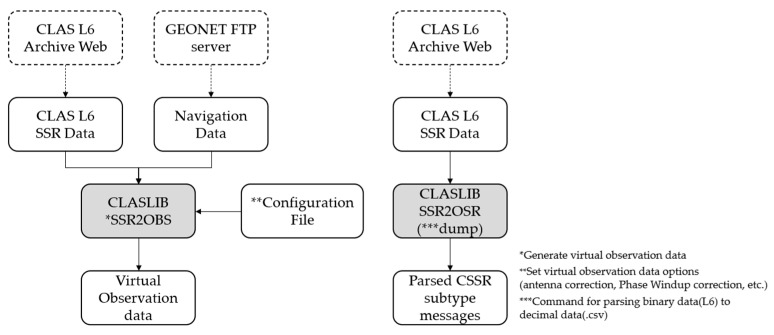
Data extraction of CLAS L6 broadcast messages using CLASLIB.

CLASLIB is an open-source library that parses the CLAS L6 messages, and the process is shown in Figure 1. It converts the CLAS L6 messages to the observation space representation of correction messages or provides parsed L6 message Type 4073 in comma-separated value formats. CLASLIB can also generate VRS-RTK GNSS observation data from the given GNSS navigation data and the corresponding L6 broadcast messages.

**Figure 2:**
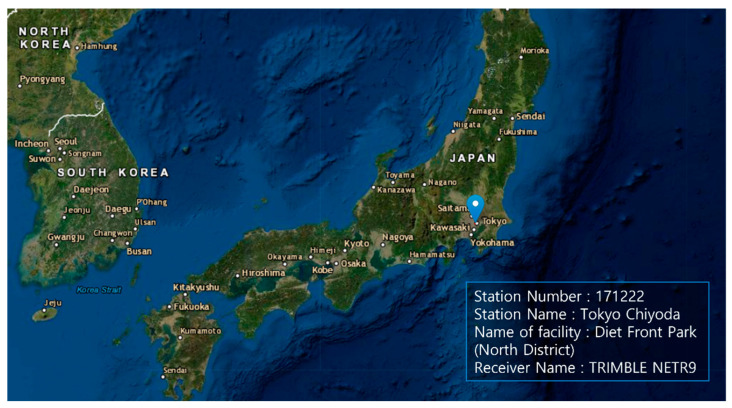
GNSS data obtained from a GEONET base station located at Chome-1 Nagatacho, Chiyoda City, Tokyo 100-0014, Japan (35.677° latitude and 139.748° longitude) [27].

**Figure 3:**
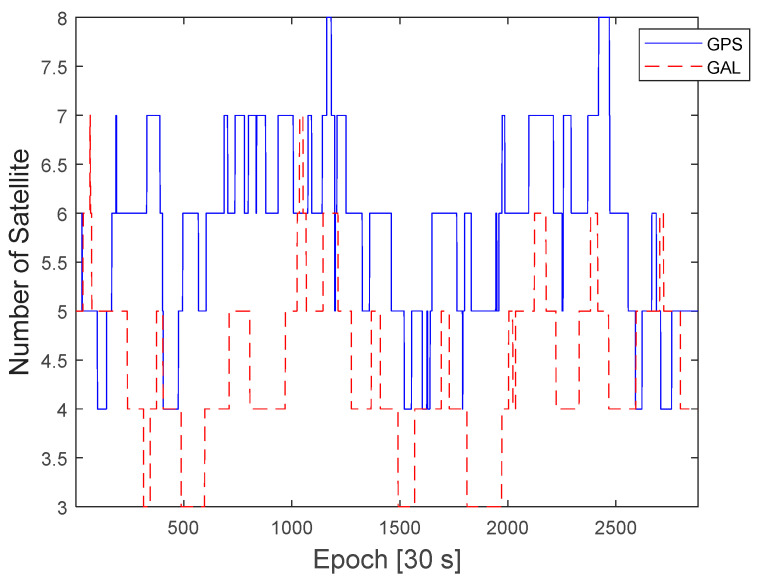
Number of visible GPS and Galileo satellites during a day in the 7-day period.

**Figure 4:**
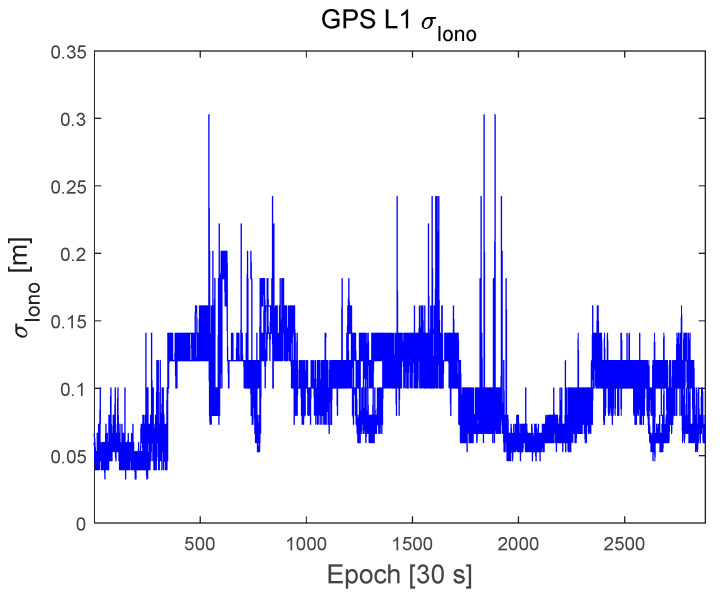
Time series of the Ionospheric correction quality, $\sigma_{\mathrm{iono}\;}$, in meters.

**Figure 5:**
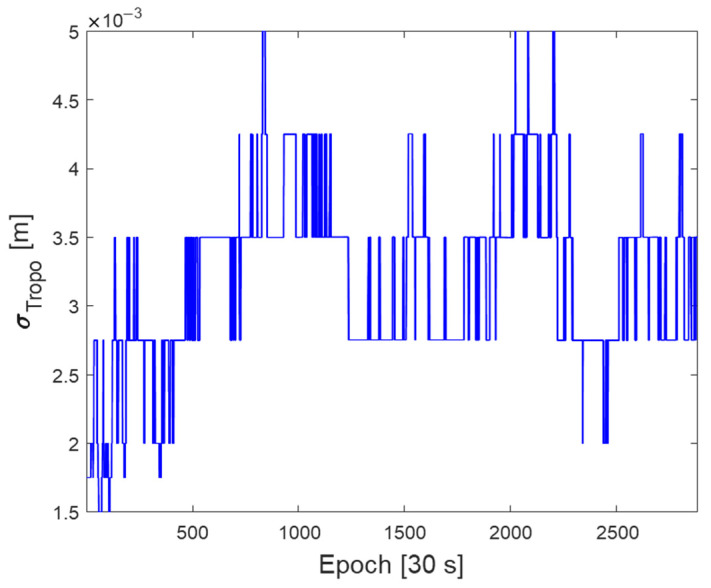
Time series of the Troposphere correction quality, $\sigma_{\mathrm{tropo}\;}$, in meters.

**Figure 6:**
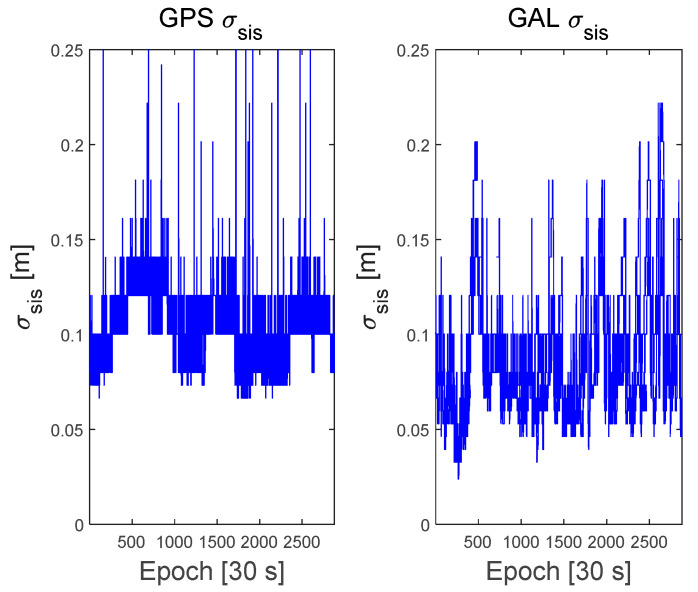
Time series of the Signal in Space correction quality, $\sigma_{sis\;}$, for GPS and Galileo satellites.

To evaluate the proposed protection-level equations, GNSS data were obtained from a GEONET base station located in Chiyoda City, Tokyo, Japan, as shown in Figure 2. The test site is located within the CLAS service volume. The GNSS dataset consisted of the GPS and Galileo RINEX observation and navigation files collected over 7 days from 00:00:00 on 11 April 2021, to 23:59:30 on 17 April 2021, with 30 s intervals. The base station uses a Trimble NETR9 receiver and a Trimble TPSCR.G5C antenna. The numbers of visible GPS and Galileo satellites during this period are shown in Figure 3. Because the satellite constellation exhibits a trend similar to that of a daily cycle, only the number of visible satellites in 1 day is shown. Figure 4 and Figure 5 show the time series of the $\sigma_{i,iono}^{}$ and $\sigma_{i,tropo}^{}$, respectively, extracted from the dataset. Figure 6 shows the time series of the $\sigma_{i,sis}^{}$ for the GPS and Galileo satellites. The user receiver multipath and noise uncertainty of the code and carrier-phase measurements are modelled as σuser,code=15sin(El) in centimeters and σuser,carrier=3sin(El) in millimeters, respectively, and are the typically used values.

### 4.2. Comparison of the VRS-RTK Position Errors and Computed Protection Levels

**Figure 7:**
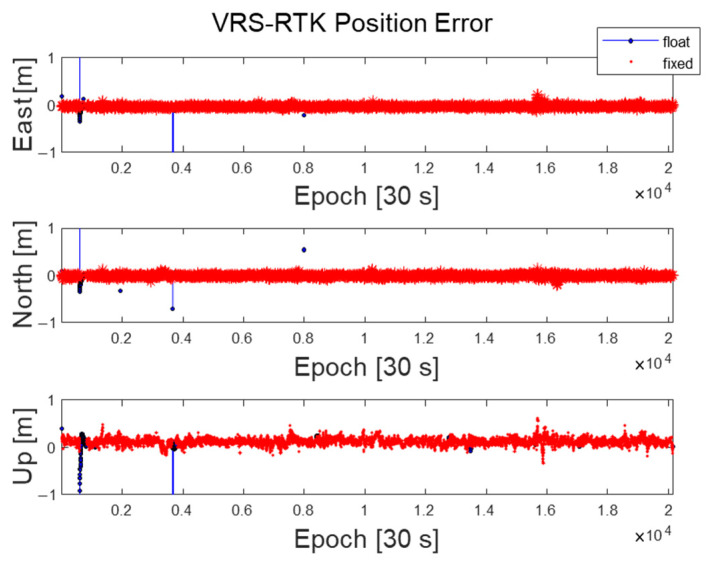
VRS−RTK position errors for float and fixed integer cases with the test dataset.

Figure 7 shows the resultant ENU position errors of zero-baseline VRS-RTK using dual-frequency GPS and Galileo satellites with the test dataset. To determine the fixed integers, a conventional test value ratio, above 3, was used. The RMS of the positioning errors with the fixed integers is 4, 2, and 14 cm in the East, North, and up directions, respectively. At certain epochs, our VRS-RTK software failed to resolve fixed integers due to cycle slips. For this particular test, 2.5% of the data contained a float solution.

**Figure 8:**
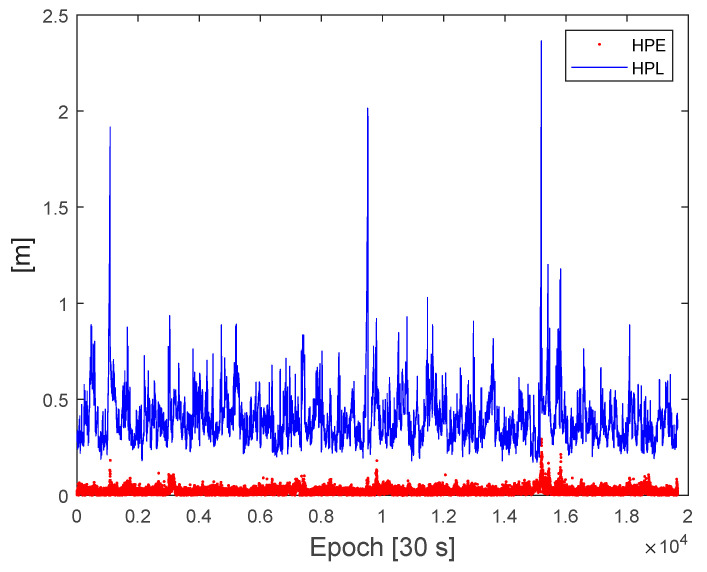
Comparison of the horizontal protection levels and horizontal position errors from the test dataset.

**Figure 9:**
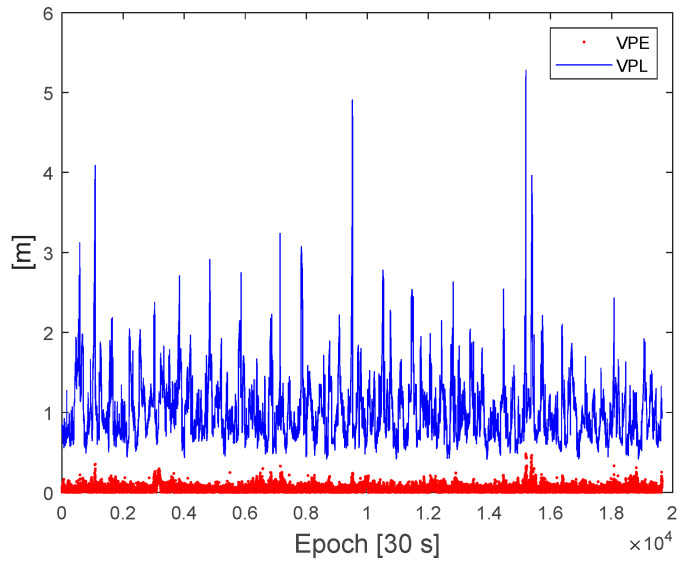
Comparison of the vertical protection levels and vertical position errors from the test dataset.

Figure 8 and Figure 9 show the computed protection levels and the position errors for the fixed integers cases with a fault-free risk probability ($I_{H0req}$) of 10^−7^. If the test value ratio was less than 3, that is, float solutions, no protection levels were computed. As shown in Figure 8 and Figure 9, all of the computed HPL and VPL were larger than the horizontal positioning errors (HPE) and vertical positioning errors (VPE), respectively. The RMS of the HPE was 4.6 cm whereas that of the HPL ranged from 25 cm to 2.3 m. The RMS of the VPE was 14 cm, whereas the VPL ranged from 50 cm to 5.2 m. At certain epochs, there are large peaks in the HPL and VPL, and these peaks occur when there is a jump in quality indices. These large protection levels occurred at very small percentages such that HPL was less than 1 m in 99.4% and VPL was less than 3 m in 99.6% of the dataset, respectively.

## 5. Discussion

The broadcast $\sigma_{i}$ of the correction messages in SBAS or CLAS is typically much larger than the actual correction error distribution because it is intentionally inflated to overbound the residual errors in range measurements such that the protection levels also overbound the position errors after applying the correction messages. In VRS-RTK, the uncertainty of the double-difference code and carrier phase measurement, $Q_{\rho }$ and $Q_{\Phi }$, respectively, is used to solve the float baseline and integer ambiguities. Then, the float integer ambiguities and their associated covariance matrices are used in the re-parameterization and search procedure for integer ambiguities. If $Q_{\rho }$ and $Q_{\Phi }$ are constructed using the broadcasted $\sigma_{i}^{}$ from CLAS, the LAMBDA process has a very large search space and a high chance of finding incorrect integer ambiguities. Therefore, the realistic values of $Q_{\rho }$ and $Q_{\Phi }$ should be used to successfully resolve the integer ambiguities in LAMBDA.

In the test results, the RMS errors in the up direction are relatively larger than in the East and North directions. The mean value of the vertical position errors had a bias of approximately 12 cm. We also observed a similar bias from zero-baseline VRS-RTK position errors with the same dataset using an open-source software tool [28]. Therefore, it is presumed that there was a bias in the reported true antenna positions of the GNSS dataset used in the test. This issue will be investigated further.

In fact, whether the receiver moves or not, protection levels can be computed in the same way as long the PPP-RTK process is adequately implemented. However, a dynamic receiver may suffer from a frequent loss and inclusion of satellites, which may overall lower the *PCF* of integer ambiguities and result in incorrect integer ambiguities. The research on this issue will also be carried out as future work.

## 6. Conclusions

This paper presents a fault-free protection level equation for the CLAS PPP-RTK service that broadcasted correction quality messages. The performance of the proposed protection level equations was tested using 7 days of the GNSS observation data and the CLAS L6 messages obtained at a base station in Tokyo, Japan, which was located within the CLAS service region. The test results with 7 days of GNSS data showed that the HPL and VPL were always larger than the HPE and VPE of the zero-baseline VRS-RTK solution. Furthermore, fault-free protection-level violations were not observed. It should be noted that most HPL and VPL values were less than 1 and 3 m, respectively. Since a rail automation, which is a very demanding problem from an integrity point of view, requires Horizontal Alert Limit of 1m [29], the proposed protection level would be able to fulfill stringent integrity and availability requirements for many applications using PPP-RTK services.

## Figures and Tables

**Table 1:** Compact SSR message type, nominal validity period, and nominal update interval.

Message Name	Message Type ID Subtype ID	Nominal Validity Period [s]	Nominal Update Interval [s]
Compact SSR Mask	MT4073,1	1	30
Compact SSR GNSS Orbit Correction	MT4073,2	60	30
Compact SSR GNSS Clock Correction	MT4073,3	10	5
Compact SSR GNSS Satellite Code Bias	MT4073,4	60	30
Compact SSR GNSS Satellite Phase Bias	MT4073,5	60	30
Compact SSR GNSS Satellite Code and Phase Bias	MT4073,6	60	30
Compact SSR GNSS URA	MT4073,7	60	30
Compact SSR STEC Correction	MT4073,8	60	30
Compact SSR Gridded Correction	MT4073,9	60	30
Compact SSR Service Information	MT4073,10	(N/A)	(N/A)
Compact SSR GNSS Combined Correction	MT4073,11	10 or 60	5 or 30
Compact SSR Atmospheric Correction	MT4073,12	60	30
